# Analysis of spleen histopathology, splenocyte composition and haematological parameters in four strains of mice infected with *Plasmodium berghei* K173

**DOI:** 10.1186/s12936-021-03786-z

**Published:** 2021-06-06

**Authors:** Huajing Wang, Shuo Li, Zhao Cui, Tingting Qin, Hang Shi, Ji Ma, Lanfang Li, Guihua Yu, Tingliang Jiang, Canghai Li

**Affiliations:** 1 Tang Center for Herbal Medicine Research, Institute of Chinese Materia Medica, China Academy of Traditional Chinese Medical Sciences, No. 16 Dongzhimen Nanxiaojie, Dongcheng District, Beijing, 100700 China; 2 Artemisinin Research Center, China Academy of Traditional Chinese Medical Sciences, Beijing, China

**Keywords:** Malaria, Spleen, Filtration, Splenocyte, *Plasmodium berghei* K173, Mouse

## Abstract

**Background:**

Malaria is a fatal disease that presents clinically as a continuum of symptoms and severity, which are determined by complex host-parasite interactions. Clearance of infection is believed to be accomplished by the spleen and mononuclear phagocytic system (MPS), independent of artemisinin treatment. The spleen filters infected red blood cells (RBCs) from circulation through immune-mediated recognition of the infected RBCs followed by phagocytosis. This study evaluated the tolerance of four different strains of mice to *Plasmodium berghei* strain K173 (*P. berghei* K173), and the differences in the role of the spleen in controlling *P. berghei* K173 infection.

**Methods:**

Using different strains of mice (C57BL/6, BALB/C, ICR, and KM mice) infected with *P. berghei* K173, the mechanisms leading to splenomegaly, histopathology, splenocyte activation and proliferation, and their relationship to the control of parasitaemia and host mortality were examined and evaluated.

**Results:**

Survival time of mice infected with *P. berghei* K173 varied, although the infection was uniformly lethal. Mice of the C57BL/6 strain were the most resistant, while mice of the strain ICR were the most susceptible. BALB/c and KM mice were intermediate. In the course of *P. berghei* K173 infection, all infected mice experienced significant splenomegaly. Parasites were observed in the red pulp at 3 days post infection (dpi) in all animals. All spleens retained late trophozoite stages as well as a fraction of earlier ring-stage parasites. The percentages of macrophages in infected C57BL/6 and KM mice were higher than uninfected mice on 8 dpi. Spleens of infected ICR and KM mice exhibited structural disorganization and remodelling. Furthermore, parasitaemia was significantly higher in KM *versus* C57BL/6 mice at 8 dpi. The percentages of macrophages in ICR infected mice were lower than uninfected mice, and the parasitaemia was higher than other strains.

**Conclusions:**

The results presented here demonstrate the rate of splenic mechanical filtration and that splenic macrophages are the predominant roles in controlling an individual’s total parasite burden. This can influence the pathogenesis of malaria. Finally, different genetic backgrounds of mice have different splenic mechanisms for controlling malaria infection.

## Background

*Plasmodium falciparum* parasites cause lethal infections worldwide, especially in Africa [1]. Reducing this disease burden continues to rely heavily on the availability and proper use of effective anti-malarial drugs. Artemisinin and its derivatives are sesquiterpene lactones with potent activity against nearly all blood stages of *P. falciparum*. There is a natural and complex variation in the pathogenesis and clinical presentation of malaria, which is influenced by host age, immunity and genetic background, as well as by environmental conditions and parasite genetics [2, 3]. Host immunity and genetic factors are estimated to account for one quarter of the total variability in malaria severity [4, 5]. Host defense mechanisms, such as removal of circulating parasites by the spleen and mononuclear phagocytic system (MPS), are thought to play a major role in rapid control of infection [6], in the presence or absence of artemisinin treatment [7].

The function of the spleen is to remove senescent red blood cells (RBCs) and circulating foreign material such as bacteria or cellular debris [8]. The structure of the spleen is complex with two overlapping blood circulations—a rapid flow by-pass, called the fast closed circulation, which accommodates roughly 90% of the splenic blood flow (100–300 mL/min in a healthy adult), and a slow open circulation in which the blood is filtered through narrow inter-endothelial slits [9, 10]. In the slow open microcirculation, RBCs navigate through the cords of the red pulp before returning to the vascular beds by squeezing between endothelial cells in the sinus walls [11–13].

Crossing splenic inter-endothelial slits poses the greatest demand on RBC deformability in the body [14] and is believed to result in the retention of less malleable RBCs or in removal of intraerythrocytic bodies. In malaria, the spleen filters infected RBCs from circulation by physical selection as well as immune-mediated recognition and phagocytosis of infected RBCs [11]. These processes play a central role in the clearance of circulating malaria parasites [6]. The rate of splenic mechanical filtration may be one factor affecting an individual’s total parasite burden and the pathogenesis of malaria. Understanding the role of the spleen in host defense may shed additional light on the variation in human susceptibility to malaria and offer insights into possible mechanisms of malaria pathogenesis.

Research on human malaria is hampered by ethical constraints that limit thorough analyses of human spleens. Thus, a rodent model of malaria infection was used here. Two outbred strains derived from the Swiss mouse, KM and ICR were used. For inbred strains, both C57BL/6 and BALB/c mice were used. The C57BL/6 mouse was bred in by 1921 C.C. Little from the Lathrop strain. Another widely used white mouse in malaria research is the BALB/c [15]. All the four mouse strain are commonly used in malaria research. Many factors can impact on the development of malaria. Among them, the mouse strain used is one of the most important factors. However, little information is available about the performance of different strains of mice when used to develop rodent models of malaria.

In the present study, the host defense against blood-stage malaria was examined by using different strains of mice infected with *Plasmodium berghei* K173 (*P. berghei* K173), a rodent-lethal strain of malaria. Parasitaemia and survival were measured to monitor the course of infection in C57BL/6, BALB/C, ICR, and KM mice. Since C57BL/6 mice were found to be more resistant to this infection, parameters indicative of a protective host response to infection were also characterized in the four strains mice. These included splenomegaly, histopathology, splenocyte subsets, haematological parameters. Here, it was observed that the rate of splenic mechanical filtration and splenic macrophages are the likely mechanisms by which an individual’s parasite burden is controlled. This can influence the pathogenesis of malaria. Finally, different genetic backgrounds of mice have different splenic mechanisms for controlling malaria infection.

## Methods

### Parasite strains and culturing conditions

*Plasmodium berghei* K173, a gift from Dr. Dai of Chengdu University of TCM, was serially passaged in vivo in mice. Infected blood was harvested at day 5–7 post-infection and stored as frozen stabilates in Alsever’s solution containing 10% glycerol.

### Mice and infection

Male C57BL/6, BALB/C, ICR, and KM wild-type (WT) mice (6–8 weeks old, weighing 18–22 g) were used in this study. Animals were purchased from Weitonglihua (Beijing, China). A total of 6 mice per group were uninfected/infected intraperitoneally with 10^7^ *P. berghei* K173-infected RBCs and were provided water and standard laboratory mouse chow diet ad libitum throughout the experiment. All mice were housed in pathogen-free animal facilities at the Institute of Chinese Materia Medica, China Academy of Chinese Medical Sciences.

### Measurement of haematologic parameters and parasitaemia

Complete blood counts were obtained with a XN-1000V [B_1_]blood analyzer (Sysmex, Japan). The percent parasitaemia was determined by counting the infected out of 1000 RBCs in random fields by microscopic counts of thin blood smears stained with Giemsa solution (Sigma-Aldrich, USA) using the formula:

$$\% {\text{ Parasitaemia}} = \frac{{{\text{Number of infected out of }}1000{\text{ RBCs}}}}{1000} \times 100$$

### Isolation of immune cells from mouse spleen

Spleen samples were surgically removed and weighed in a sterile hood. One part of each spleen sample was removed and fixed in 4% paraformaldehyde for histopathologic examination, and the remainder was used for isolation of splenocytes.

Spleens harvested under aseptic conditions were ground into small pieces and passed through a sterilized 200 mesh screen to prepare crude splenocyte suspensions at room temperature. Samples were then centrifuged at 1000 revolutions per minute (rpm) for 8 min (min) at 4 °C, and the remaining splenocyte suspension was re-suspended in red blood cell Lysis Buffer (Thermo Fisher Scientific, USA). After a 10 min treatment, 1× phosphate buffer saline (PBS) was added to dilute the samples, and then centrifuged at 1000 rpm for 8 min at 4 °C. The pelleted splenocytes in each group were washed twice and adjusted to concentrations of 5 × 10^6^ cells/mL with 1× PBS.

### Analysis of splenocyte subsets

The single cell splenocyte suspensions were stained with the following anti-mouse antibodies: Brilliant Violet 510 anti-mouse CD45 (Biolegend, Cat 103138), FITC Anti-Mouse CD3(17A2) (Proteintech, Cat 51000626), PerCP/Cyanine5.5 anti-mouse CD4 (Biolegend, Cat 100434), APC Anti-Mouse CD8a (53-6.7) (Proteintech, Cat 51000549), and PE anti-mouse F4/80 (Biolegend, Cat 123110). Splenocytes were incubated with monoclonal antibodies in the dark for 30 min at 4 °C. According to the manufacturer’s instructions, the specificity of labelling was confirmed by isotype-matched antibody staining controls. The labelled cells were analysed using a CytoFLEX flow cytometer (Beckman coulter, USA).

### Histological examination

Spleen tissues were fixed in 4% paraformaldehyde, dehydrated through graded alcohol, embedded in paraffin, sectioned at a thickness of 3 μm, and then stained with haematoxylin & eosin (H&E) and Giemsa solution according to standard procedures. Then, the stained slides were mounted in neutral balsam and covered with coverslips. Histopathologic changes were observed by light microscopy (BX43F Olympus, Japan).

### Statistical analysis

Data were analysed using SPSS 19.0 (IBM, USA) and reported as mean ± standard deviation (SD). Significant differences between groups were analysed using one-way ANOVA, and are designated as follows: *p < 0.05, **p < 0.01 and ***p < 0.001 relative to the uninfected control groups. Survival curves were calculated using GraphPad Prism 8.0 (GraphPad Software, USA).

## Results

### Progression of infection with *Plasmodium berghei* K173 in mice

**Fig. 1:**
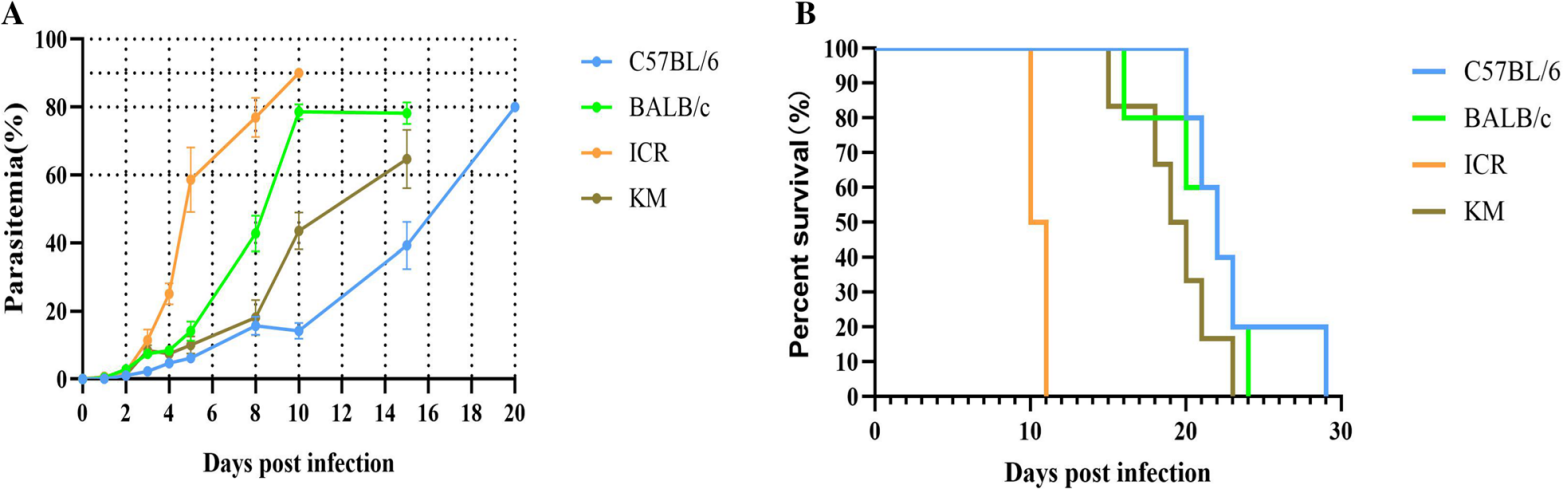
Parasitaemia and survival curves in C57BL/6, BALB/c, ICR and KM mice following *P. berghei* K173 infection. **A** The percentage of parasitaemia is presented as the arithmetic mean of each mouse strain ± SD. **B** Survival of mice without treatment (n = 6) as a function of days post infection (dpi) with *P. berghei* K173

The combination of different mouse strains and parasites resulted in different disease outcomes following infection. BALB/c, ICR and KM mice developed infection with parasites observed in the blood as early as 1 day post infection (dpi), whereas C57BL/6 mice converted at 2 dpi. The clinical course of disease severity was progressive up to the point of death in all infected groups (Fig. 1A). On 5 dpi, the parasitaemia in ICR mice was 58.6%, 90% at 10 dpi, and all animals died between 9 and 11 dpi (Fig. 1B). In BALB/c mice, parasitaemia approached 50% by 8 dpi, and 80% on 10 dpi. The BALB/c mice all succumbed between 16 and 24 dpi. On the 15th day, the highest parasitaemia of the KM mice was 65%, and the animals all died between 15 and 23 dpi. The parasitaemia of C57BL/6 mice was close to 50% on the 17th day, and the parasitaemia reached 80% on the 20th day. All C57BL/6 mice died from malaria by 29 dpi.

It was observed that C57BL/6 mice were more resistant to infection than the other strains examined, and had a longer survival time. The lethal parasitaemia of KM mice was the lowest observed, but its survival was shorter than that of C57BL/6 mice. The lethal parasitaemia of ICR mice was higher than other strains, although these mice succumbed the fastest.

### Haematological parameters

In this study, RBC counts (mean ± SD, 10^12^/L) for uninfected control mice were as follows: C57BL/6 (10.01 ± 0.25), BALB/c (7.93 ± 1.01), ICR (8.16 ± 0.16), KM (8.64 ± 0.29). These values are within normal ranges, as reported previously [16]. By 5 dpi, all strains of mice presented with anaemia, thrombocytopenia, and leukocytosis (Table 1).

**Table 1:** Effects of *P. berghei* K173 infection on some haematological parameters in infected mice on 5 dpi

Haematological parameters	C57BL/6		BALB/c		ICR		KM	
	uninfected	Infected	uninfected	Infected	uninfected	Infected	uninfected	Infected
PLT (10^9^/L)	1278.83 ± 89.60	285.00 ± 34.88↓***	1310.50 ± 57.76	312.33 ± 19.59↓***	1635.67 ± 68.14	296.00 ± 33.80↓***	1476.17 ± 99.99	341.33 ± 31.87↓***
RBC (10^12^/L)	10.01 ± 0.25	7.75 ± 0.62↓***	7.93 ± 1.01	6.62 ± 0.37↓*	8.16 ± 0.16	3.39 ± 0.35↓***	8.64 ± 0.29	4.44 ± 0.34↓***
HGB (g/L)	149.67 ± 2.66	113.83 ± 8.45↓***	132.33 ± 12.07	101.50 ± 4.42↓*	137.17 ± 4.26	60.00 ± 6.96↓***	129.67 ± 5.98	68.83 ± 4.92↓***
HCT (%)	47.23 ± 0.96	37.78 ± 3.03↓***	40.40 ± 3.28	32.58 ± 1.33↓*	42.48 ± 1.10	21.27 ± 2.19↓***	42.67 ± 2.02	24.77 ± 1.86↓***
MCV (fL)	46.52 ± 0.43	48.40 ± 0.71↑***	45.02 ± 0.67	49.22 ± 1.48↑*	51.77 ± 1.33	62.72 ± 3.15↑***	48.75 ± 0.83	56.26 ± 1.12↑***
MCH (pg)	14.71 ± 0.07	14.56 ± 0.24	14.73 ± 0.15	15.46 ± 0.63↑*	16.86 ± 0.35	17.55 ± 0.76	15.18 ± 0.35	15.75 ± 0.12↑*
MCHC (g/L)	316.83 ± 2.04	301.17 ± 2.14↓***	327.17 ± 4.35	310.50 ± 5.17↓***	321.67 ± 3.26	283.83 ± 5.49↓***	310.67 ± 3.38	279.00 ± 5.66↓***
RDW-CV (%)	17.35 ± .26	16.20 ± 0.83↓*	19.03 ± 0.99	16.72 ± 0.92↓*	15.85 ± 0.32	19.97 ± 1.21↓*	16.75 ± 0.57	15.83 ± 1.64
RDW-SD (fL)	22.80 ± 0.59	24.50 ± 1.16↑*	25.82 ± 2.22	26.87 ± 1.49	26.70 ± 0.84	41.11 ± 4.13↑***	27.27 ± 0.74	31.98 ± 1.85↑***
WBC (10^9^/L)	2.74 ± 0.39	5.84 ± 2.16↑*	4.37 ± 0.93	8.64 ± 1.95↑*	2.52 ± 0.35	5.63 ± 1.64↑*	1.65 ± 0.30	3.45 ± 0.43↑***
NEUT (%)	10.13 ± 0.4	88.33 ± 5.83↑***	32.68 ± 9.50	76.53 ± 10.66↑***	16.50 ± 1.07	91.65 ± 2.59↑***	14.78 ± 1.31	94.77 ± 1.04↑***
LYMPH (%)	82.80 ± 2.78	5.71 ± 3.13↓***	52.68 ± 9.16	---	73.98 ± 1.77	---	74.57 ± 2.09	---
MONO (%)	6.35 ± 1.55	5.68 ± 2.45	2.10 ± 0.38	7.00 ± 2.46↑*	4.66 ± 0.85	2.71 ± 0.85↓*	4.85 ± 1.01	3.11 ± 0.46↓*
EO (%)	1.47 ± 0.67	0.47 ± 0.38↓*	2.13 ± 0.59	1.33 ± 0.68	4.06 ± 0.53	3.98 ± 2.73	4.60 ± 1.66	0.71 ± 0.24↓*
BASO (%)	0.32 ± 0.23	0.63 ± 0.23	0.16 ± 0.05	0.38 ± 0.04↑*	0.25 ± 0.20	0.45 ± 0.08	0.51 ± 0.17	0.58 ± 0.20

Values are given as mean ± SD (n = 6). * and *** indicate *p* < 0.05 and *p* < 0.001 compared with the uninfected group, respectively. “---” indicates that the blood analyser did not obtain the haematological parameters

*PLT* platelet, *RBC* red blood cell, *HGB* haemoglobin, *HCT* haematocrit, *MCV* mean corpuscular volume, *MCH* mean corpuscular haemoglobin, *MCHC* mean corpuscular haemoglobin concentration, *RDW-CV* RBC distribution width coefficient of variation, *RDW-SD* RBC distribution width standard deviation, *WBC* white blood cell, *NEUT* neutrophil, *LYMPH* lymphocyte, *MONO* monocyte, *EO* eosinophil, *BASO* basophil

Malaria infections induce lymphocytopenia and an increase in neutrophils, which is indicative of systemic inflammation [17]. In all four strains of mice, the percentages of lymphocytes decreased compared to the baseline values (Table 1). In all mice, the percentages of neutrophils increased relative to uninfected control mice (Table 1). On 5 dpi, the percentage of monocytes in BALB/c (7.00 ± 2.46, *p* < 0.05) mice had increased. In contrast, monocyte counts decreased in ICR (2.71 ± 0.85, *p* < 0.05) and KM (3.11 ± 0.46, *p* < 0.05) mice. The percentage of monocytes in C57BL/6 (5.68 ± 2.45) mice was not significantly different from that of the uninfected group (Table 1).

### Gross and histopathologic analysis of the spleen

**Fig. 2:**
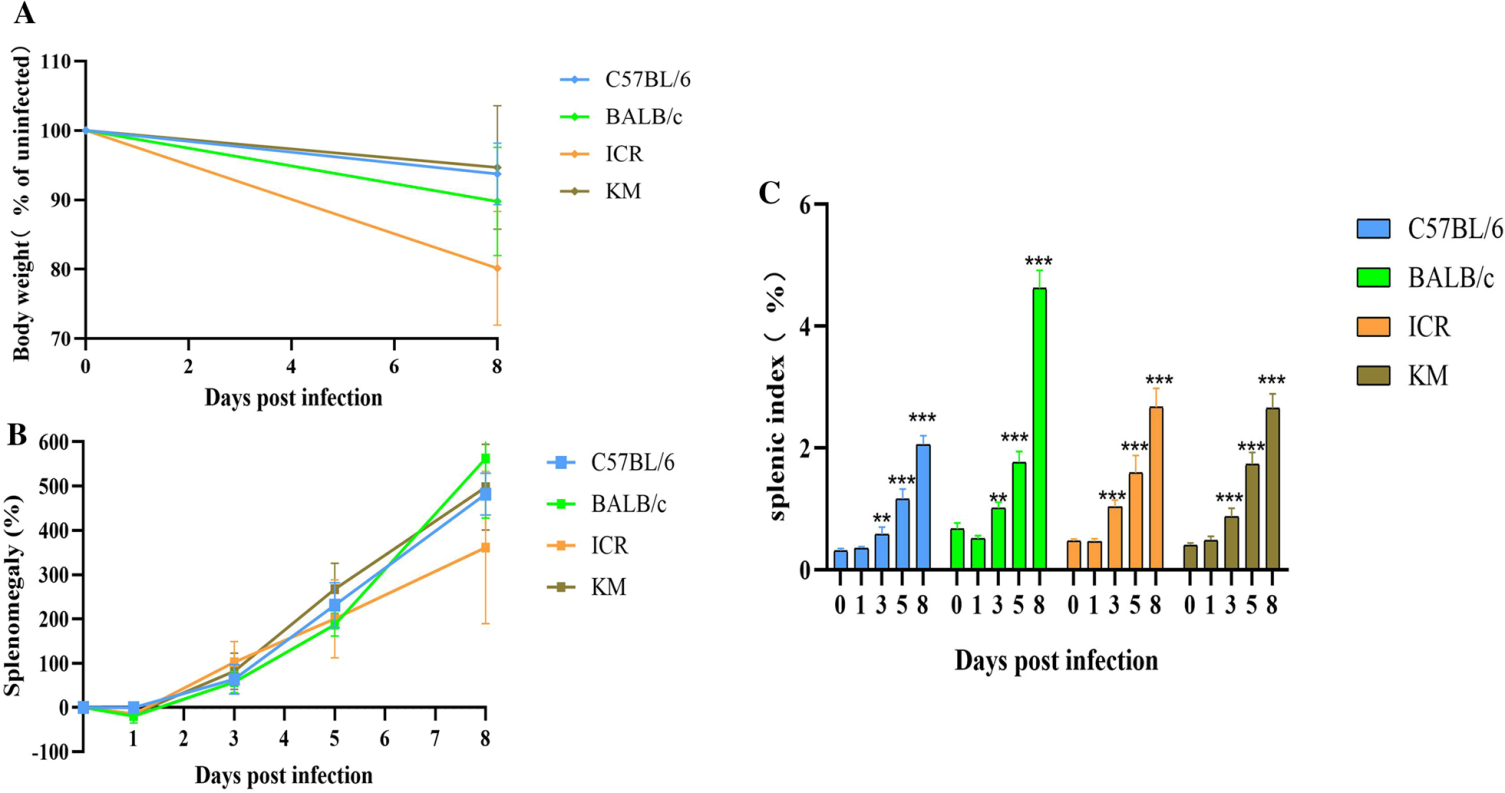
The change in body weight (**A**) and spleen weight (**B**) in the four strains of mice, spleen weights were normalized to the weight of uninfected groups (%). **C** Splenic index in uninfected and *P. berghei* K173 infected mice. Spleen weights of infected mice were determined on days 3, 5, and 8 post infection. The splenic index was determined as the ratio of spleen weight to body weight. All data are presented as mean ± SD, n = 6. ** and *** indicate statistical significance at* p* < 0.01 and *p* < 0.001 compared with the uninfected group, respectively

The spleen is an important site of erythropoiesis, the clearance of infected RBCs (iRBCs), and immune system activation in response to blood-stage malaria [18]. In the present study, the body weight of infected mice was observed to decline following infection with *P. berghei* K173 (Fig. 2A). The spleen weight of infected groups was observed to increase beginning at 3 dpi (Fig. 2B). In response to infection, all mice experienced significant splenomegaly, but the splenic index was significantly higher in infected BALB/c mice (Fig. 2C).

**Fig. 3:**
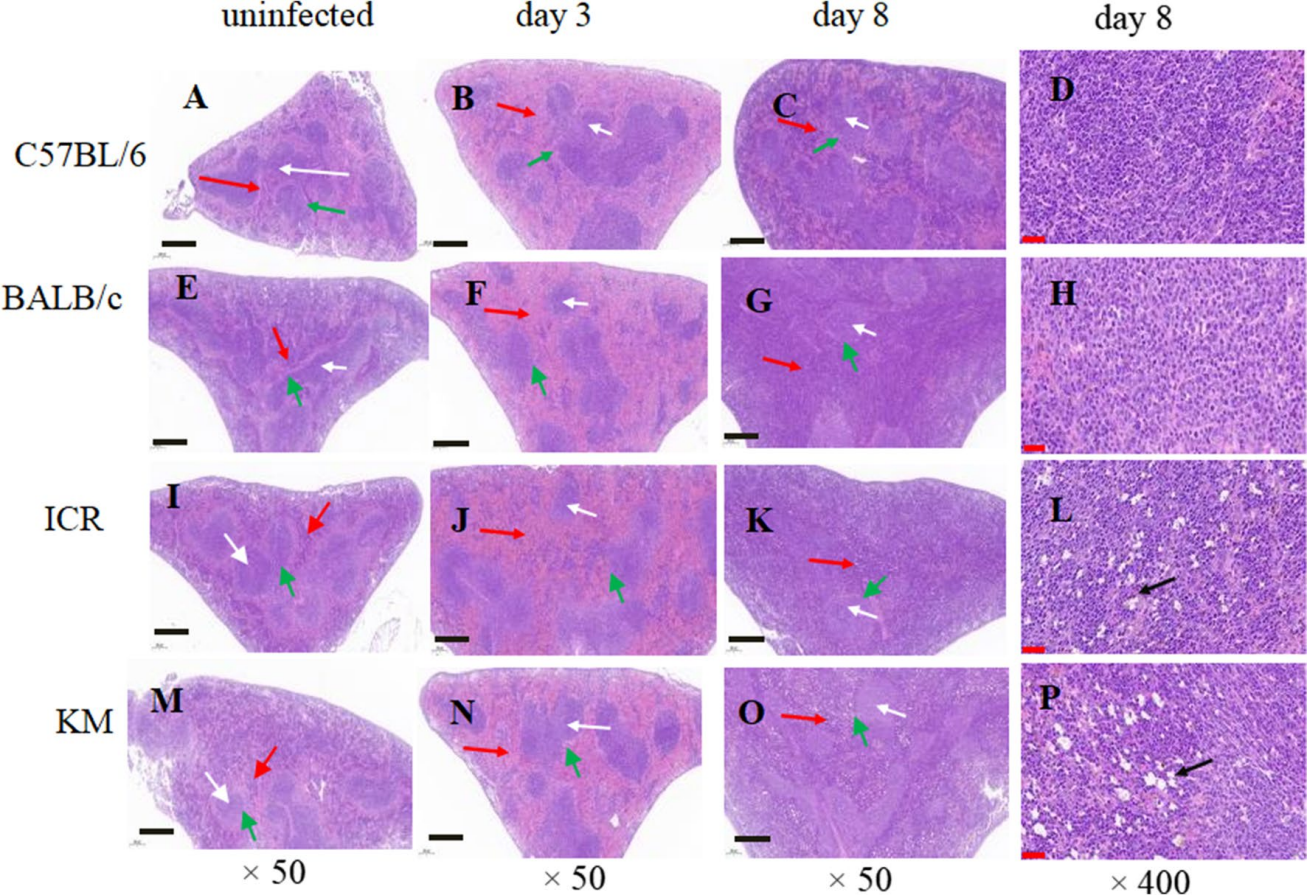
H&E staining sections of spleen from infected or uninfected mice (n = 6). white arrows: white pulp; red arrows: red pulp; green arrows: marginal zone; black arrows highlight vacuolation; black scale bars represent 400 μm; red scale bars represent 40 μm

Figure 3 shows the histopathological sections of the spleen tissue. A clear distinction between the red and white pulp, resting follicles, and marginal zones were evident in the spleen of normal uninfected control mice (Fig. 3A, E, I, M). Severe congestion and enlarged red pulp were observed in spleens of infected mice on 3 dpi (Fig. 3B, F, J, N). Increases in red pulp cellularity, the structure of the white pulp was destroyed and the clear marginal zones surrounding follicles became inapparent in spleens of infected mice on 8 dpi (Fig. 3C, G, K, O). Furthermore, extensive vacuolation in the red pulp at 8 dpi was observed in spleens from ICR and KM mice (Fig. 3L, P).

**Fig. 4:**
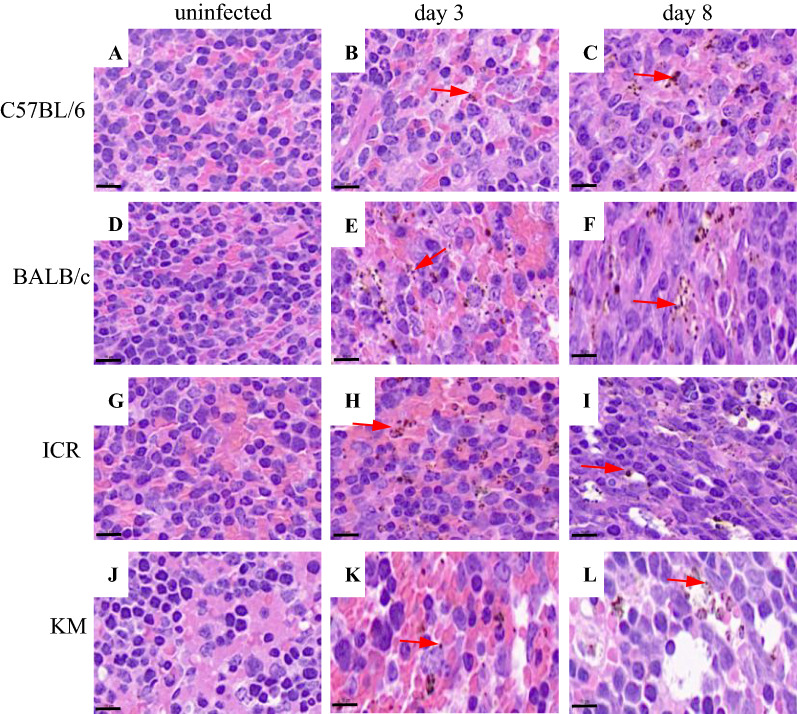
H&E staining of spleen from control and *P. berghei* K173 infected mice (n = 6). All the images represent ×1000 magnification, and scale bars represent 10 μm. Red arrows indicate malarial pigments, which appear as small brown punctate staining

Spleen sections were prepared and examined via bright-field microscopy coupled with haematoxylin and eosin (H&E) staining (Fig. 4). In all *P. berghei* K173 infected mice, parasite pigments (red arrows) in the pulp histiocytes and sinusoidal lining cells were observed in contrast to the uninfected mice (Fig. 4A, D, G, J). The extent of malaria pigmentation in the spleen is correlated with high parasitaemia. The spleens of C57BL/6, BALB/c, ICR, and KM mice filtered late trophozoite stages as well as a fraction of earlier ring-stage parasites out of the blood at 3 dpi (Fig. 4B, E, H, K). However, KM mice only retained the late trophozoite stage on day 8 (Fig. 4I). This phenomenon may be due to changes of the splenic structure, which could have resulted in alterations to the filtering function of the spleen. Transformations in the red pulp and splenic vasculature may modulate the mechanical retention threshold and regulate the microcirculatory trapping of blood cells in the spleen.

### Analysis of splenocyte subsets

**Fig. 5:**
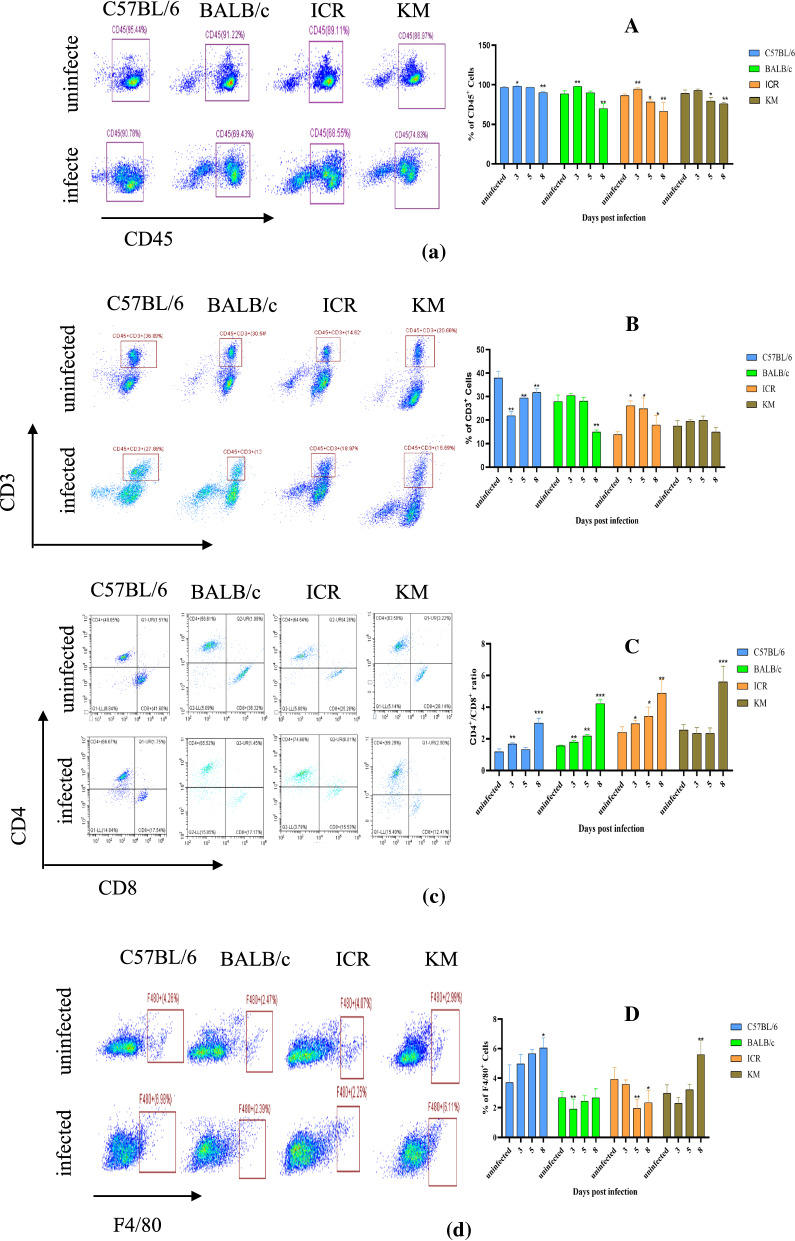
Flow cytometry analysis of splenocyte subsets at 3, 5, 8 dpi. Splenocytes of mice infected with parasites were incubated with the required antibodies according to the manufacture's protocol for antibody dilution, incubation duration, and were analysed in a CytoFLEX flow cytometer. *, ** and *** indicate statistical significance at *p* < 0.05, *p* < 0.01 and *p* < 0.001 compared with the uninfected group, respectively (Data are presented as the mean ± SD, n = 6). **a** Representative dot plots of splenocytes gated for CD45^+^. Percentages in the right quadrant indicate the frequencies of CD45^+^ cells within total splenocytes. **b** Representative dot plots of CD3^+^ splenocytes expressing CD45^+^. Percentages in the upper right quadrant indicate the frequencies of CD3^+^ cells within total CD45^+^ cells. **c** Representative dot plots of CD4^+^ and CD8^+^ gated on CD45^+^CD3^+^ splenocytes. Percentages in the upper left quadrant indicate the frequencies of CD4^+^ cells within total CD45^+^CD3^+^ cells, percentages in the lower right quadrant indicate the frequencies of CD8^+^ cells within total CD45^+^CD3^+^ cells. **d** Representative dot plots of F4/80^+^ splenocytes. Percentages in the right quadrant indicate the frequencies of F4/80^+^ cells within total splenocyte

Next, the distribution of macrophage and T lymphocyte subpopulations were analysed. Using single-cell suspensions from *P. berghei* K173 infected or uninfected control spleens, flow cytometry was performed to quantify total leukocytes (CD45^+^ cells), total T lymphocytes (CD45^+^CD3^+^ cells), T cell subsets (CD4^+^ and CD8^+^ cells), and monocytes/macrophages (F4/80^+^ cells) (Fig. 5). At 8 dpi, a significant decrease in total leukocyte in both C57BL/6, BALB/c, ICR and KM infected mice (*p* < 0.01) was observed (Fig. 5A). The data also indicated a significant decrease in the percentages of the T lymphocytes (CD3^+^ cells, except ICR and KM infected mice) (Fig. 5B). The ratio of CD4^+^/CD8^+^ indicated a significant increase (Fig. 5C) in all infected mice.

The trends in macrophage percentages differed between the four strains of mice. The percentage of macrophages did not change significantly in BALB/c infected mice. A decrease in the percentage of macrophages was however observed in the infected ICR mice. Conversely, a significant increase in the percentages of the macrophages in C57BL/6 and KM infected mice as compared to uninfected mice was observed (Fig. 5D).

Splenic red pulp macrophages, located between the splenic cords and venous sinuses, are well positioned to clear iRBCs and are important for controlling blood-stage malaria [19]. In the present study, after infection with *P. berghei* K173, the percentages of macrophages in the spleen of C57BL/6 mice exhibited the greatest increase of all the mouse strains (Fig. 5D), and the parasitaemia progressed the slowest compared to the other mouse strains (Fig. 1A).

The percentage of macrophages in the spleen of KM mice was higher than uninfected group on 8 dpi. The spleens in KM mice exhibited structural disorganization and remodelling (Fig. 3P), which likely affected the mechanical retention threshold (Fig. 4L). As a result, only late trophozoite stages were retained, which led to a significantly higher parasitaemia in KM than C57BL/6 mice. The percentages of macrophages in ICR infected mice were lower than uninfected mice (Fig. 5D), and the parasitaemia was higher than other strains during the course of *P. berghei* K173 infection (Fig. 1A).

These data show that the rate of splenic mechanical filtration and the splenic macrophages may be important factors in determining an individual’s total parasite burden and potentially influencing the pathogenesis of malaria, and different genetic backgrounds of mice have different mechanisms for controlling malaria infection in the spleen.

## Discussion

Host defense mechanisms are central to rapid control of malaria infection [6], in the presence or absence of artemisinin treatment [7]. The rate of splenic mechanical filtration may be one factor affecting an individual’s total parasite burden and the pathogenesis of malaria. The inbred C57BL/6 and BALB/c mouse, and outbred KM and ICR mouse strains are widely employed to study malaria. However, little information is available about the role of the spleen and its functioning in different mouse strains and the rate of malaria disease progression.

In this study, the survival time of mice infected with *P. berghei* K173 varied, although the infection was uniformly lethal. Since the ICR mice developed disease rapidly and died quickly, this model may be suitable for the study of acute or severe malaria. Although the onset time of BALB/c mice was earlier than that of KM mice, the survival time of BALB/c mice was not significantly different from that of KM mice. The lethal parasitaemia of KM mice was 65%, while the lethal parasitaemia of other strains was over 80%. The growth rate of C57BL/6 mice was slower than that of other strains, and the survival period was longer than that of other strains.

The spleen is a key organ for removal of parasitized red blood cells, generation of immunity and production of new red blood cells during malaria. The importance of the spleen for the control of malaria was confirmed by studying the response of splenectomized humans and rodents to infection. Humans with acute *P. falciparum* malaria who had previously undergone splenectomy had decreased clearance of iRBCs from the circulation [20]. The mice subjected to partial splenectomy presented a level of parasites similar to that of non-splenectomized mice, while the animals subjected to full splenectomy had twice the amount of circulating parasites [21]. Furthermore, parasite clearance after drug treatment was delayed in splenectomized patients, with RBCs containing dead parasites being retained in the circulation for prolonged periods, compared with individuals with a functional spleen [22].

The spleen eliminates infected erythrocytes occurs through activation of cellular and humoral immune responses, and through mechanical filtration. White and red pulp structures have specific functions in the human spleen. The white pulp is a major control center for the humoral immune response, especially to circulating antigens. The red pulp exerts a unique and subtle control of the surface integrity and biomechanical properties of erythrocytes. To be left in circulation, RBCs must be fit enough to cross a very specific structure of red pulp sinuses, the inter-endothelial slit (IES). Older erythrocytes, or those modified by innate or acquired conditions, are eventually retained in the splenic red pulp and processed by red pulp macrophages (RPMs) [23].

During asexual replication (including the sequential ring, trophozoite, and schizont stages), parasite maturation induces changes in the host RBC with novel proteins synthesis [24, 25]. As the parasite develops, the infected RBC loses its biconcave shape and progressively becomes spherical and rigid [26]. Furthermore, the surface area-to-volume ratio decreases, the shear elastic modulus of the plasma membrane, and the cellular viscosity increase [27]. The loss of RBC deformability is not limited to mature stages, but starts soon after parasite invasion. During the ring stage (within the first 16–24 h after RBC invasion), iRBC undergo up to 9.6% surface area loss [28, 29]. The altered deformability of the *Plasmodium*-infected RBC may result in increased retention in the spleen. More than 50% of ring-iRBC are retained upon ex vivo transfusion through human spleens [29]. These retention and accumulation processes stem from the splenic screening of RBC deformability [30]. However, no direct evidence exists demonstrating the correlation among the rate of splenic mechanical filtration, macrophages, and infection severity. In this study, at 3 days post *P. berghei* K173 infection, malaria pigments were observed in the red pulp in great abundance. The pigments consisted of parasites in the ring and trophozoite stages.

During acute attacks of malaria, splenomegaly is one of the typical signs of malaria, and the degree of splenomegaly often impacts the host’s ability to mount a successful response to the parasite [31]. Besides an increase in the organ volume and mass, the spleen also exhibits structural disorganization and remodelling. These changes include expansion of the red pulp, transient loss of the marginal zone, increased vasculature, and activation of barrier cells, which may establish a blood-spleen barrier that can drastically alter splenic blood circulation [13, 32, 33]. In this study, the spleen index of infected groups was observed to increase from 3 dpi. Severe congestion and enlarged red pulp was evident in the infected mice. By 8 dpi, infection-induced increases in red and white pulp cellularity and the marginal zones surrounding follicles disappeared in all strains of mice examined. However, the spleens of C57BL/6 and BALB/C infected mice maintain their structural integrity, although the spleen index of BALB/C changed significantly. The spleen of ICR and KM mice exhibited severe vacuolation, and the splenic structure was highly atypical, with many of the features absent at this time. This could be a result of the spleen structures of mice with different genetic backgrounds possessing different tolerances and pathologies in infection with malaria.

The comparison of haematological parameters in mice infected with *P. berghei* K173, different mouse strains showed that only the change trends in the percentages of monocytes was observed. Cells of the monocyte/macrophage lineage are one of the main sources of cytokines in malaria-infected individuals [34]. Monocytes recognize *P. falciparum* biological products and *P. falciparum*-infected erythrocytes directly through pattern recognition receptors (PRR) [35], as well as complement- or IgG-opsonized erythrocytes and parasite components via complement receptors and Fcγ receptors [36]. Activated monocytes have several important effector functions in the host defense against malaria, including phagocytosis [37], cytokine production [38], and modulation of adaptive immune responses [34, 39]. At 5 dpi, compared with the uninfected groups, the percentage of monocytes in BALB/c mice was elevated, whereas the values in ICR and KM mice were reduced. Conversely, the parasitaemia in ICR mice was significantly higher than KM mice. The relative abundance of monocytes in infected C57BL/6 mice did not change, although this strain was most effective at controlling parasitaemia.

At 5 dpi, the number of macrophages in the spleen of ICR mice infected with *P. berghei* K173 was lower than uninfected controls, and the parasitaemia was higher than other strains. At 8 dpi, the number of macrophages in the spleen of C57BL/6 mice infected with *P. berghei* K173 was higher than that in uninfected controls, and the parasitaemia was lower than other strains. The percentage of macrophages in the spleen of infected ICR mice was lower than the uninfected group, and the parasitaemia increased rapidly. During the infection period, the ratio of macrophages in the spleen of BALB/c mice was not significantly different from that of the uninfected group. The growth rate of the parasitaemia was lower than that in ICR mice, but higher than that of C57BL/6 mice. In a systemic pathological study of cerebral malaria in African children, enlarged spleens and abundant malaria pigments in splenic macrophages were observed in the majority of the 103 fatal cases [40]. These observations point to an important role of the spleen in parasite control. In this experiment, the parasitaemia was inversely proportional to the percentage of spleen macrophages, which may be explained as macrophages complement the mechanical filtration of the spleen to control parasitic infections.

## Conclusions

In the present study, four widely used mouse strains, C57BL/6, BALB/c, KM, and ICR were utilized to study pathogenesis of *P. berghei* K173. The survival time of mice infected with *P. berghei* K173 varied. ICR mice developed disease rapidly and died quickly. BALB/c mice showed clinical signs associated with malaria later than ICR mice, which were characterized by weight loss and lethargy. The parasitaemia growth rate of C57BL/6 mice was slowly than that of other strains, and the survival period was longer than other strains. The parasitaemia of KM mice never exceeded 65%.

Compared with the uninfected groups, all strains of mice infected with *P. berghei* K173 had splenomegaly. At 3 days post *P. berghei* K173 infection, malaria pigments were observed in the red pulp in great abundance. At 8 dpi, the spleen of ICR and KM mice exhibited severe vacuolation, and the splenic structure was highly atypical, suggesting that the spleen structures of mice with different genetic backgrounds possessing different tolerances and pathologies to infection with malaria.

Analysis of splenocyte subsets, revealed an inverse proportional relationship between the percentage of spleen macrophages and parasitaemia, which may be explained as macrophages complementing the filtering function of the spleen to control parasitic infections.

## Data Availability

The datasets used and/or analysed during the current study are available from the corresponding author on reasonable request.
